# Discovering compassion in medical training: a qualitative study with curriculum leaders, educators, and learners

**DOI:** 10.3389/fpsyg.2023.1184032

**Published:** 2023-06-28

**Authors:** Charles B. Lane, Erin Brauer, Jennifer S. Mascaro

**Affiliations:** ^1^Department of Family and Preventive Medicine, Emory University School of Medicine, Atlanta, GA, United States; ^2^Department of Spiritual Health, Emory University Woodruff Health Sciences Center, Emory Healthcare, Atlanta, GA, United States

**Keywords:** medical education, well-being, compassion, burnout, healthcare, training

## Abstract

**Background:**

Compassion is considered a fundamental human capacity instrumental to the creation of medicine and for patient-centered practice and innovations in healthcare. However, instead of nurturing and cultivating institutional compassion, many healthcare providers cite the health system itself as a direct barrier to standard care. The trend of compassion depletion begins with medical students and is often attributed to the culture of undergraduate medical training, where students experience an increased risk of depression, substance use, and suicidality.

**Objectives:**

This qualitative study aims to develop a more comprehensive understanding of compassion as it relates to undergraduate medical education. We used focus groups with key stakeholders in medical education to characterize beliefs about the nature of compassion and to identify perceived barriers and facilitators to compassion within their daily responsibilities as educators and students.

**Methods:**

Researchers conducted a series of virtual (Zoom) focus groups with stakeholders: Students (*N* = 14), Small Group Advisors (*N* = 11), and Medical Curriculum Leaders (*N* = 4). Transcripts were thematically analyzed using MAXQDA software.

**Results:**

Study participants described compassion as being more than empathy, demanding action, and capable of being cultivated. Stakeholders identified self-care, life experiences, and role models as facilitators. The consistently identified barriers to compassion were time constraints, culture, and burnout. Both medical students and those training them agreed on a general definition of compassion and that there are ways to cultivate more of it in their daily professional lives. They also agreed that undergraduate medical education – and the healthcare culture at large – does not deliberately foster compassion and may be directly contributing to its degradation by the content and pedagogies emphasized, the high rates of burnout and futility, and the overwhelming time constraints.

**Discussion:**

Intentional instruction in and cultivation of compassion during undergraduate medical education could provide a critical first step for undergirding the professional culture of healthcare with more resilience and warm-hearted concern. Our finding that medical students and those training them agree about what compassion is and that there are specific and actionable ways to cultivate more of it in their professional lives highlights key changes that will promote a more compassionate training environment conducive to the experience and expression of compassion.

## 1. Introduction

Compassion, the affectionate motivation to help alleviate suffering, is crucial to the purpose, function, and structure of health systems (Lown, 2014; Sinclair et al., 2016; Cochrane et al., 2019; Trzeciak and Mazzarelli, 2019). Neurobiologically distinct from empathy, compassion builds on the empathic concern that can arise in response to suffering and includes the motivation and intention for improving the well-being of another (Singer and Klimecki, 2014). Compassion can lead to profound benefits for the individual experiencing compassionate motivation, the recipients of compassionate actions, and to the systems that support their emergence (Trzeciak and Mazzarelli, 2019). Moreover, patients and their families, providers, and health organizations identify compassion as a hallmark of quality care (Paterson, 2011; Sinclair et al., 2016; Cochrane et al., 2019; Trzeciak and Mazzarelli, 2019). For patients, physician compassion is linked with improved clinical outcomes (Hojat et al., 2011; del Canale et al., 2012), greater patient satisfaction (Wang et al., 2018), and adherence to treatment (Neumann et al., 2007). Of benefit to health systems, compassionate physicians order fewer unnecessary tests and referrals (Bertakis and Azari, 2011), commit fewer major errors (West et al., 2009), and are less likely to be sued for malpractice (Moore et al., 2000). For the provider, the experience of compassion is associated with positive affect (Stellar et al., 2015), pro-social motivation (Mascaro et al., 2013), improved resilience (Klimecki et al., 2014), and has even been proposed as an antidote to burnout (Singer and Klimecki, 2014; Neff and Dahm, 2015; Trzeciak and Mazzarelli, 2019).

Despite the beneficial qualities of compassion, the professional and organizational cultures of allopathic medicine have increasingly adopted policies and positions that create barriers to compassionate care, rather than facilitating it (Shanafelt et al., 2019). Excessive preauthorization and documentation, staff shortages, focus on relative value units and patient volume, and limited appointment lengths contribute to disturbing rates of provider/physician depression, substance abuse, and even suicide, all of which likely disturb a provider’s capacity for compassion (Sprang et al., 2007; Lancet, 2019; Shanafelt et al., 2019; The National Academy of Medicine, 2019; Trzeciak and Mazzarelli, 2019). Moreover, even though they are buffered from the burden of clerical medicine, there is a measurable atrophy of compassion among medical students (Hojat et al., 2009). Quickly after matriculation into medical training, students exhibit similar rates of burnout, depression, substance use, and suicide as the physicians training them (Ludwig et al., 2015; Jackson et al., 2016; The National Academy of Medicine, 2019; Dyrbye et al., 2021). It is imperative that deliberate steps are taken to inure future providers from the degradation of compassion and overwhelming burnout so pervasive in their chosen career path (Neumann et al., 2011). Undergraduate medical education (UME) offers a unique opportunity to operationalize compassion interventions and curricula designed to entrain sustainable compassion. Medical students spend a large portion of their training away from the bedside, in the relative safety and structure of classrooms and simulations. Additionally, compared to residents and faculty, students have limited and purposefully regulated responsibility for patient care; their primary responsibility is to learn (Liaison Committee on Medical Education, 2022). Developing the tools and skills required to maintain their compassion and resilience as they grow intimate with human suffering and the successes and failures of medicine would benefit their own well-being, the patients they serve, and the teams they will lead. Yet, despite the evidence for the value of compassion, two recent systematic reviews found that current approaches to compassion training are of limited duration, designed primarily for developing individual compassion rather than institutional, and created as adjuncts to the core curriculum rather than as an integrated, longitudinal, multi-faceted approach to compassion cultivation (Patel et al., 2019; Sinclair et al., 2021a). These approaches can be overly burdensome to students and teachers and are often less effective than curricula that incorporate a systematic culture of compassion (Michalec, 2009; Neumann et al., 2011).

We hypothesize that making compassion cultivation a central part of the content and pedagogies utilized in undergraduate medical training would produce profound benefits for medical students, those training them, and ultimately the healthcare systems and patients they serve. In order to develop a more comprehensive approach to compassion cultivation in medical training, we conducted a series of focus groups with medical students and educators to query their conceptions of compassion. We sought to identify how key stakeholders in undergraduate medical education describe compassion, as well as the factors that facilitate and inhibit the enaction of compassion in their day-to-day professional environment.

## 2. Materials and methods

### 2.1. Study overview

To address our aim, we conducted a series of virtual (Zoom) focus groups with key stakeholders: small group advisors (SGA), medical curriculum leaders (MCL), and medical students (MS) between October 2021 and January 2022. The study was conducted in accordance with approval from our university’s institutional review board and all study participants provided informed consent.

### 2.2. Participants

We used convenience sampling to recruit between 4 and 12 participants per focus group. To facilitate conversation and to better ensure that stakeholders felt comfortable and safe to share their responses (Ayala and Elder, 2011), focus groups were subdivided by participant role in medical education: medical students (*n* = 14), small group advisors (SGAs; *n* = 11), and medical curriculum leaders (MCLs; *n* = 4). SGAs planned a professional development seminar to accommodate the focus groups and MCLs were recruited by direct email. Students were recruited by listserv emails sent to all currently enrolled MD students and offered a $15 gift card for their participation. Our inclusion criteria consisted of the current school of medicine students and faculty; we had no exclusion criteria. To ensure that SGAs and MCLs felt safe to share their beliefs and to better ensure the anonymity of these small populations, we did not acquire demographic data for these groups; MS demographics data were collected by Qualtrics survey during recruitment and are summarized in Table 1.

**Table 1:** Medical student demographics.

Age	20–23	2
	24–27	9
	28–31	3
Gender	Cis Female	10
	Cis Male	4
Ethnicity	Black or African American	4
	Hispanic or Latin	1
	White or Caucasian	7
	Asian or Pacific Islander	2
Years in Medical school	<1 year	4
	1+ year	4
	2+ years	1
	3+ years	4
	4+ years	1
Years Since Completing Undergrad	<1 year	2
	1+ year	3
	2+ years	4
	3+ years	2
	4+ years	3

### 2.3. Focus groups

Focus groups queried stakeholders’ general beliefs about compassion using an interview guide that was created prior to the focus group sessions. We asked the participants about their conceptions of the nature of compassion, whether they conceived of compassion as sustaining or draining, whether and how compassion can be cultivated, and what supports or hinders their experience of compassion in their day-to-day responsibilities. We reached saturation with available educators and held an additional focus group with students to confirm the saturation of themes.

### 2.4. Analysis

Focus groups were recorded, transcribed, and the transcripts were then anonymized. We used MAXQDA.20 to manage and thematically analyze the transcripts. We first familiarized ourselves with the data by reviewing the interview guide and all transcripts to generate a preliminary codebook of themes identified by researchers. Then, two researchers (CL and EB) independently used this preliminary codebook to thoroughly review the transcripts and identify any salient themes missing from the codebook, to reduce redundancy of themes, and establish any identified hierarchical grouping. After this, the entire research team discussed codes to reconcile any coding differences, to ensure concordance and reliability, and to finalize the codebook. The finalized codebook was used to generate reports in MAXQDA.20 for which the results are described below.

## 3. Results

### 3.1. Defining compassion

Assessing student and educator conceptions of compassion revealed striking similarities in the descriptions across the three stakeholder groups. Emergent themes included that compassion is more than empathy (themes and representative quotes found in Table 2). All stakeholder groups characterized compassion as an emotional arousal that is more than empathy and typified by motivated action to address another’s suffering. A second emergent theme was that compassion is ineffable. All stakeholder groups acknowledged a felt somatic sense of compassion that they reported could not be described by words and made the experience of compassion feel more impactful to them. A third theme was that compassion can be cultivated. There was unanimous agreement across all focus groups that compassion is not a fixed trait, but rather something that can be cultivated and grown with age and experience.

**Table 2:** Defining compassion.

Theme	Representative quote
Compassion is more than empathy	“So, I think [compassion is] similar to empathy, but I think it implies more of an active role, maybe. That it entails perceiving the suffering of others, but not just perceiving, but being moved by it, being inspired by it, being pushed to do something about it.” (MS)
	“I think when I hear the term compassion, it has a connotation of action for me. Like I am, in addition to putting myself in someone else’s shoes, there’s some action that I am taking to alleviate their suffering or whatever they are experiencing. And so that to me, is one of the key differences that sets compassion apart [from empathy] is because there’s an action element.” (SGA)
Compassion is ineffable and somatic	“I think compassion maybe is I feel for you. Like basically you are going through something does inspire something inside of me. And I may not understand completely, but I feel for you, is compassion.” (MS)
	“I think there’s a little bit more to it in terms of the sort of inexpressible, indescribable, emotional state that I feel when I see somebody who’s hurting, that is a little bit bigger” (SGA)
	“I feel like compassion is something that comes really deep from within. I can be very empathetic to a patient, but to really feel what they are going through, I do not know if I could really describe it. It’s, you are almost going through the journey with them and you feel it almost on a physical level.” (MCL)
Compassion can be cultivated	“I think like compassion can be learned with knowledge and like, I feel like people’s beliefs about things can always change over time. And the more you learn, the more you can, you can grow your compassion.” (MS)
	“I also feel like compassion is something that I think sort of naturally grows with age. I mean, in healthy aging and healthy social development.” (MS)
	“I also think you can improve upon compassion and teach people ways to show it and maintain it under stress.” (SGA)
	“I also think it has something to do just with life experience and not only just in medicine, but just living. As, you know, just getting more appreciation for the human condition. And so, I think there’s definitely a capacity to increase one’s compassion.” (SGA)

SGA = small group advisor, MS = medical student, MCL = medical curriculum leader.

### 3.2. Positive influences on compassion

Participants identified factors that facilitate the enaction of compassion in their day-to-day professional environment (themes and representative quotes found in Table 3). Again, there was a significant overlap in answers across stakeholder groups. The importance of self-care and meeting one’s basic needs to maintain compassion emerged as a central theme in every focus group. Participants discussed self-care in terms of attending to their own needs, including adequate sleep, self-regulation, therapy, spiritual practice, and spending time with a nurturing community of colleagues and loved ones. All focus groups also identified the importance of life experiences, which exposed them to diverse people and helped build cognitive empathy for others’ perspectives, as supportive of compassion. For students, this theme extended to the experiential learning they received in their medical training. They acknowledged that their medical training challenged their compassion when they spent most of their time behind computers and studying for written exams. Rather, it was the experiential learning opportunities where they felt compassionate intentions arise. Finally, students were more likely than educators to express the importance of compassionate role models and teachers to cultivate compassion in their training, often providing specific exemplars of compassion.

**Table 3:** Positive influences on compassion.

Theme	Representative quote
Self-care	“It’s hard to be compassionate when you are completely zapped of all your energy, and not getting to take care of yourself really well.”(MS)
	“Because we get busy and we just go, I find myself just running in and out of rooms and, you know, not really pausing to take that time for my own compassion for myself to even eat or do anything…how do we improve the compassion for ourselves? Because if we do that, we are going to be more compassionate toward others.” (SGA)
	“Well, I think burnout is definitely a thing. And then maybe projects that are motivated initially by compassion can get warped. You can sort of forget about your initial motivations, but I think that actually speaks to the importance of these sort of daily rituals and practices of reminding yourself of that meaning so that you do not get burnt out.” (MS)
	“I’m thinking [compassion] is closely aligned with juggling too many things… And so, kind of always working in that space where you feel like am I with integrity, able to actually take on the various roles that I’m being asked to do.” (MCL)
Life experiences	“Having lived in other cultures for a significant number of years in my life, I became accustomed to looking at things through other people’s eyes. And as an educator, that’s what I consider my responsibility to be, is to get people to look at things through other people’s eyes. The system of licensing physicians does not encourage that. Convincing medical students that they should do that…is what my job is all about” (MCL)
	“I think that one of the big things that changes our ability to empathize or feel compassionate for other people is about lived experience. And sometimes when somebody is different than us and we have no perceived connection to that person, then it’s very hard to feel a sense of connection with them. But as you interact with a person as you build shared community or shared connection or you just have sometimes really like life overturning experiences, [it] will just really change your perspective on something. And I think those can all change how you interact with and perceive other people’s situations.” (MS)
	“I do not think my compassion has increased and simultaneously decreased as much in my life as, as it has been in med school…those times of just like studying, all we are doing is like book stuff and lectures and whatnot, I think it’s a lot easier to lose compassion. But then it’s moments like certain moments at OPEX, or even like my CLSM or something. I think experience is a lot more, experience is, I think experience is definitely a lot more powerful of a teaching tool.” (MS)
Role models	“Well, one thing that just came to mind that helps cultivate compassion. I think in the, I do not know if it’s the day-to-day thing, but I really am impressed by the chaplains that work at the hospital. So, getting to shadow the chaplains is something that I’ve taken advantage of and found to be extremely powerful. And just to see things from their perspective.” (MS)
	“I had a pretty extraordinary OPEX [out-patient experience] preceptor. Who taught me a lot about the way I want to speak to patients and how I want to be and treat my team. She modeled compassion for herself, for her patients, for the whole, like the nurses, the RT’s, the clinical researchers. Like she was so, so impactful that I went to OPEX every week instead of every other week, because I loved her that much. And I think it was really great to get that experience, especially as an M1/M2 cause I was like, wow, like this can be amazing.” (MS)
	“I almost think that the most, like what’s been most helpful for me in terms of like, seeing how compassion can be done in medicine is by example.” (MS)

SGA = small group advisor, MS = medical student, MCL = medical curriculum leader.

### 3.3. Negative influences on compassion

Participants also identified factors that inhibit the enaction of compassion in their day-to-day professional environment (themes and representative quotes found in Tables 4, 5). Emergent themes regarding the hindrances to compassion were slightly more varied between stakeholder groups. Differences were highlighted in their discussion regarding the culture of medical education. Students primarily described how the culture, content, and pedagogies of medical education were more likely to drain their compassion than bolster it. They discussed an overemphasis on book learning and multiple-choice exams, as well as experiencing a toxic culture on the wards. SGAs, on the other hand, were more likely to describe instances of burnout, futility, and disrespect as barriers to their compassionate intentions. They expressed a sense of hopelessness and exhaustion when their efforts to support patient care and student development confronted the limitations and failures of the health system and undergraduate medical licensing requirements. SGAs also expressed that disrespectful behavior created a barrier to their compassionate motivations. Whether it came from patients, colleagues, or students, and regardless of whether it was directed at them, their colleagues, or public health efforts in general, incivility was consistently noted as a barrier. Finally, all groups acknowledged time constraints as a major barrier to their experience of compassion. This included having time to complete professional responsibilities, time to take care of themselves, time to spend with colleagues and loved ones, as well as enough time to teach and learn the requisite content prior to graduation.

**Table 4:** Negative influences for students.

Theme	Representative quote
Culture, content, and pedagogies of medical education	“I think medicine is a very interesting field where it’s very focused on compassion, but I think there’s also a lot of pressure on us to perform at the highest level in like all these different things. And to be very competitive and to be very on top of our game and academic and all these things. And I think that kind of culture takes away, oftentimes, from our ability to actually be compassionate to our patients. And so I think fundamentally the culture does not actually support it, even though we say that that is like an important part of medicine.” (MS)
	“I’m like, people learn more from seeing. If medical students are going to be taught about compassion, and when you go on the wards, all you see is non-compassionate people around you. You’d be more likely to forget all you have learned and really, yeah. I think people really, I mean, such a human thing to be like the people you are with most of the time. So I guess I’m wondering how that compassion will spread to the people who are teaching medical students on the wards, and not just 18 months that we get in class.” (MS)
	“I think those times of just like studying all we are doing is like book stuff and lectures and whatnot. I think it’s a lot easier to lose compassion, and all those other, I guess, kindness type traits.” (MS)
	“We sit through the bioethics lectures in class and they are just like this weird detached abstract theory. I’m just like, I do not have time for this. This is this is it’s borderline pointless sometimes. And when you are not paying attention to like real people that you are going to serve 1 day and all you are doing is sitting in lecture and whatever, if you overemphasize any one thing, man that’s, that’s exhausting” (MS)
	“[There is] an opportunity for our generation of physicians to do better, that like people do not go through their education with traumatizing experiences from someone who’s screaming at them for being incompetent, when like the point is that we are incompetent because we are trying to learn. And I am sure that nobody has gone through this without having an experience like that, but maybe like we can be better. And maybe that’s the point. Yeah, it’s tough.” (MS)
Time constraints	“I think it feels like a med school with, you know, all the things are crammed into it, just seems at odds with like living a life of compassion in some senses because like most of the time I feel like a lot of us do not even have enough time and room to be compassionate towards ourselves, our friends who live in different states, our family and stuff like that. A lot of outside relationships sort of get pushed to the side for a lot of people. And then like in that sense, it’s hard to have compassion.” (MS)
	“I think just like time is like a big factor a lot of times as a barrier to compassion. I’m thinking about when I was on internal medicine, I had a patient who was really struggling and I ended up spending a decent amount of time every day, just talking to her about random things. It was just 30 min every morning. It was really nice…it’s not even like medical care, you know. I am literally just sitting with her…I could tell she really appreciate it. And I really enjoyed it as well. And then as the rotation got busier, that became less and less possible.” (MS)
	“I think it feels like med school – you know, all the things are crammed into it – just seems at odds with like living a life of compassion in some sense. Because, like most of the time I feel like a lot of us do not even have enough time and room to be compassionate towards ourselves, our friends who live in different states, our family and stuff like that. A lot of outside relationships sort of get pushed to the side for a lot of people. And then like in that sense, it’s hard to have compassion.” (MS)

MS = medical student.

**Table 5:** Negative influences for faculty.

Theme	Representative quote
Burnout, futility, and disrespect	“Becoming overly frustrated with systems can definitely impact my ability to feel compassion.” (SGA)
	“When you have a huge number of very needy patients and you sort of take all of them on, it’s really hard to- because you in some ways carry that burden a little bit yourself also. And I find that if I were seeing 10 half days of clinic a week, I do not think I would have enough left of me to keep giving to my patients and take care of myself.” (SGA)
	“I mean it, you know, people’s lives are really hard. And when you have got five people in a row that are maybe in abusive situations, or maybe homeless, or you just see no way out for them and you are doing the best that you can, and you are trying to find supports that do not exist for them. And they want to live and they want to do well. And you know that you have medication that could help them do well, but there’s 40 other things in the way of that. And you are trying to figure that out, but those, those supports do not exist. I mean, and you feel those people’s pain and it’s really tragic.” (SGA)
	“I think probably the biggest piece for me is disrespect towards me or towards other people of a group that I’m working with…I can feel sort of a wall coming up that makes it more challenging for me to be compassionate.” (SGA)
Time constraints	“I think the students feel often, some students I’m talking to, feel overwhelmed with the amount of things [they] are being asked to take on or learn or feel deeply engaged in at one time. And it becomes impossible, right, to actually be committed to all of those things in the way one has perhaps envisioned themselves to be.” (SGA)
	“I think time is a really big one for me. And I know that, outside of fatigue or stress, that if I start to feel that I am running very short on time, for example in clinic, I think I get worse at showing compassion.” (MCL)
	“I think we have to take the time and have the opportunity to know each other. On somewhat of a personal level, a little bit.” (SGA)
	“Time. I mean the four-letter word always. Right. And I do not, I mean, definitely with patients…and same thing with students, you know. Just life, you know, it’s hard to sit there and say, okay, I’m going to invest. And once I do, once I stop myself and say, okay, this is about the other person, everything else is like, well, how important are those other things anyway, but yeah, I think time is the biggest. Time.” (MCL)

SGA = small group advisor, MCL = medical curriculum leader.

## 4. Discussion

Compassion is central to quality health care and antithetical to harmful states of burnout that so many healthcare professionals experience. Although medical education offers a formative time for sustainable compassion training, rather than preparing medical students for the rigors of practicing medicine, many students do not receive the training and support necessary to maintain their compassionate motivations in the face of personal and health systems limitations, often with devastating consequences (Hojat et al., 2009). Beyond measurable declines in compassion, within a short time of starting training, students experience increases in burnout, substance use, depression, and death by suicide (Ludwig et al., 2015; Jackson et al., 2016; The National Academy of Medicine, 2019; Dyrbye et al., 2021). Moreover, studies examining the root causes of both the increase in mental distress and the decline in compassion point to the larger culture of medicine and have done so for decades (Pence, 1983; Neumann et al., 2011; The National Academy of Medicine, 2019). Rather than promoting the individual and collective benefits afforded through the expression of compassion, medical students are quickly inculcated with a mindset of “perfectionism, lack of vulnerability, and low self-compassion” (Shanafelt et al., 2019).

With the ultimate goal of facilitating the development of a comprehensive compassion curriculum for undergraduate medical education, this study examines how medical students, junior and senior small group advisors, and deans and directors of medical curricula conceive of compassion in the medical training environment. We also explored these key stakeholders’ perceptions of how their day-to-day routines and responsibilities support or hinder their compassion. When participants define compassion in their own terms, there is remarkable concordance across both educators and students with the definition commonly found in the scientific literature (Goetz et al., 2010; Trzeciak and Mazzarelli, 2019; Mascaro et al., 2020). Across all focus groups, participants described compassion as an ineffable motivation that is more than empathy, demands action, and can be cultivated. Participants’ understanding that compassion is more than empathy is consistent with what has been identified as contributing to compassion’s apparent buffering effects to empathic distress and burnout (Singer and Klimecki, 2014; Trzeciak and Mazzarelli, 2019). Additionally, their identification of the ineffable felt sense of compassion is consistent with the physiological regulatory effects associated with compassion, including changes in stress and immune responses (Pace et al., 2010; Stellar et al., 2015). Just as anger can be felt in our bodies as tightness in our jaw, increased heart rate, or a sense of agitation, compassion has a somatic experience that researchers are uncovering is associated with widespread physiologic benefits (McCraty and Zayas, 2014; Stellar et al., 2015; Kirby, 2017; Volynets et al., 2020). Regarding the prospect of potential intervention, previous research has identified the belief that compassion is not a fixed trait – which was unanimous in our study population – as a prerequisite for effective compassion training (Sinclair et al., 2021b).

With respect to the factors of their day-to-day responsibilities that support their compassion, participants consistently identified the importance of life experiences. For students, they valued the experiences that put them in contact with others, but contrarily shared how they spend most of their time dedicated to over-simplifications in lieu of humanistic, context-dependent, whole-system science. Educators, on the other hand, talked more about the experiences they or their students may have had before coming to medical school “in the real world,” (i.e., not necessarily during formal medical training). For all groups, although self-care was seen as conducive and necessary for their compassion, participants also acknowledged how self-care was anathema to medical culture. The apparent importance of role models for student compassion highlights the harmful downstream effects of a system in which so many of their mentors and educators are burned out, under-resourced, and limited in their own compassionate reserve (Shanafelt et al., 2019). It follows from this that one of the best ways to cultivate sustainable compassion among medical students is to support their educators.

Confirming the above limitations to supporting student and educator compassion during undergraduate medical training, participants pointed overwhelmingly to the “status quo” as an active barrier to their day-to-day compassion. For students, this barrier emerged in relation to general lecture and test formats, specific clinical and non-clinical learning opportunities, and certain behaviors of potential mentors. Compounding this sentiment was a sense that there was not enough time in the day to accomplish all their academic demands, let alone attend to their personal well-being. For educators, a sobering and honest discussion of burnout and their sense of futility when fighting a poorly incentivized health system made clear that maintaining their compassion is often a practice of sheer individual will. Without institutional support, educators expressed how disrespect could challenge their ability to be compassionate towards others, regardless of circumstances. This sentiment also highlights a potential impact of the COVID-19 pandemic on our results, as many educators shared how the increased demand on the healthcare infrastructure stressed their already limited bandwidth, leaving little room for a compassionate understanding of those who are impolite, disregard public health measures, or endanger others. Again, time constraints permeated every discussion with educators and contributed to a sense of hopelessness among SGAs and MCLs.

This study had several limitations. Beyond the small sample size of this study, the general limitations of convenience sampling create the potential for sampling bias. Focus groups should allow for a natural flow of conversation, and the resulting probing questions varied slightly from group to group. Additionally, while this study targeted UME, medical students and their formal educators are not siloed. The perspective of graduate medical education and continuing medical education, as well as the beliefs of nurses, pharmacists, social workers, support serves, administrators, industry, and patients, would help further elucidate the role of compassion in training future physicians. Additionally, future work can build upon the findings from this research by examining conceptions of compassion held at other institutions to examine whether our findings generalize to other undergraduate medical schools and to the broader medical training landscape. Continuing this work will be critical to developing a comprehensive approach to cultivating compassion within medical education and sustaining an effort to mend the professional culture of healthcare.

## 5. Conclusion

Medical students, those training them, and current patients are desperate for programs and interventions that prioritize and facilitate compassion in healthcare. Given the widespread benefits for those receiving, those expressing, and even those promoting compassion, it seems imperative that prompt and deliberate actions are taken to cultivate compassion in all aspects of medicine. To help with that endeavor, this research identifies the day-to-day facilitators and barriers to compassion in medical training. The sample is small, and it would be appropriate to continue studying the issue with a larger sample. Further research can develop more granular, actionable steps to inculcate the foundation of medical instruction with a culture conducive to creating the next generation of physician. Armed with sustainable compassion, these future leaders in medicine can persevere in greater numbers and help promulgate compassionate care for themselves, their colleagues, and their patients.

## Data availability statement

The raw data supporting the conclusions of this article will be made available by the authors, without undue reservation.

## Ethics statement

The studies involving human participants were reviewed and approved by Emory University Institutional Review Board. The ethics committee waived the requirement of written informed consent for participation.

## Author contributions

CL conceived the study, helped develop the interview guide, IRB proposal, and initial code book, co-led focus groups, transcribed the recordings, and coded the transcripts. EB provided feedback on the interview guide, IRB proposal, and codebook. She co-led focus groups, coded the transcripts, and provided substantive feedback on the manuscript. JM secured the grant proposal that funded this work and provided guidance and oversight on the IRB proposal, interview guide, conduct of the focus groups, analysis, and interpretation. She also provided substantive feedback on several drafts of this manuscript. All authors contributed to the article and approved the submitted version.

## Funding

This work was supported by the National Center for Advancing Translational Sciences of the National Institutes of Health, under Award Number UL1TR002378.

## Conflict of interest

The authors declare that the research was conducted in the absence of any commercial or financial relationships that could be construed as a potential conflict of interest.

## Publisher’s note

All claims expressed in this article are solely those of the authors and do not necessarily represent those of their affiliated organizations, or those of the publisher, the editors and the reviewers. Any product that may be evaluated in this article, or claim that may be made by its manufacturer, is not guaranteed or endorsed by the publisher.
